# Effects of Fortified Laying Hen Diet with *Moringa oleifera* Leaves and Goji Berries on Cholesterol and Carotenoid Egg Content

**DOI:** 10.3390/foods11203156

**Published:** 2022-10-11

**Authors:** Maria Maisto, Fortuna Iannuzzo, Elisabetta Schiano, Roberto Ciampaglia, Angiola Labanca, Domenico Montesano, Vincenzo Piccolo, Pasquale Rossi, Gian Carlo Tenore

**Affiliations:** 1 Department of Pharmacy, University of Naples Federico II, Via Domenico Montesano 59, 80131 Naples, Italy; 2 Sud Rienergy s.r.l. Agricultural Company, Via Favella Della Corte, 87064 Corigliano Calabro, Italy

**Keywords:** goji berries, *Moringa oleifera* leaves, functional eggs, cholesterol content, carotenoids, laying hen

## Abstract

The biofortification of basal laying hen feed with natural matrices can improve the beneficial potential of eggs produced without relying on artificial fortification. The present study aimed to evaluate the effects of hen diet supplementation with dried Moringa leaves (DML) and goji berries (DGB) on egg functional properties in terms of cholesterol and carotenoid content. Forty Lohman Brown Classic laying hens were randomly divided into four groups. The control group (G1) received the basal poultry diet, group G2 received a diet with 5% DML + 10% DGB, group G3 received a diet with 3% DML + 7% DGB, and group G4 received a diet with 15% DML. HPLC-DAD analysis showed that feed supplementation positively influenced the egg carotenoid content, with a valuable increase in xanthophylls concentration, especially lutein (+333.24% in G4, +258.15% in G2, +189.24% in G3, compared to G1). The same trend was also followed by the β-carotene concentration (+181.38% in G3 and +116.01% in G4, compared to G1). Furthermore, the eggs obtained from G3 showed the lowest cholesterol content (−47.08%). Additionally, the performed antioxidant assays showed maximum activity in G2 (+39.11 compared to G1 for the DPPH test) and in G4 (+31.11 compared to G1 for the ABTS test). In conclusion, the G2 experimental diet could be potentially used in poultry industries to produce “functional eggs”.

## 1. Introduction

Eggs have a relevant role in well-balanced human nutrition due to their considerable amount of essential nutrients [1]. This food product is a precious source of not only polyunsaturated fatty acids (PUFA) but also vitamins, minerals, and especially carotenoids [2]. Specifically, carotenoids contained in eggs are concentrated mainly in the yolks, the most representative of which are lutein, β-carotene, zeaxanthin, and canthaxanthin [3]. Among egg carotenoids, carotenes have a low accumulation efficiency in the yolks, while xanthophylls (lutein, zeaxanthin, canthaxanthin) reach a valuable concentration due to their hydrophilicity [4]. Chemically, xanthophylls have a higher polarity than carotenes (due to the hydroxylations of benzyl groups), which results in favorable pharmacokinetic parameters [5]. Considering their beneficial effects in humans, lutein, and zeaxanthin, are generally defined as macula pigments (MP) due to their specific accumulation in the macula region of the human retina. To date, the treatment with MP represents a first-choice strategy for the management of age-related macular degeneration (AMD) [6]. In addition, these molecules have shown remarkable activity in the prevention of cardiovascular diseases (CVD), strokes, cancer, and neurodegenerative diseases in humans [7].

Beyond such valuable health effects, the carotenoid concentration in egg yolks also has a commercial implication [8]. Consumers typically prefer a more intense coloration of yolks, which depends on their carotenoid concentration. It is well established that the chemical composition of animal feed may influence the quality of a hen’s egg yolk, and intense yolk color may be achieved by the use of feed enriched with carotenoids [9]. Since these molecules also contribute to improving the health of hens and chickens [10], they represent a desirable and even necessary part of the feed. Currently, the majority of commercial eggs are produced by supplementing the fodder for hen farms with commercially synthesized carotenoid mixtures [11]. To overcome the use of artificial carotenoids, the same natural matrices, rich in carotenoids, may be added to the feed production as functional ingredients, which are able to increase the carotenoid concentration [12,13]. If, on the one hand, eggs are a valuable source of protein in an equilibrate and healthy diet, on the other hand, their consumption has to be controlled due to their cholesterol content. To date, according to a meta-analysis study, egg consumption is not directly associated with the risk of CVD and cardiac mortality in the general population. However, egg consumption may be associated with an increase in the incidence of type 2 diabetes among the general population and CVD comorbidity among diabetic and hypercholesterolemic patients [14].

*Moringa Oleifera* is claimed to be the miracle plant or the tree of life [12]. This recognition refers to its extraordinary content of phytochemicals in every single part of the plant. Specifically, Moringa leaves are largely used for different purposes, such as an alternative food source to contrast infant malnutrition [15], forage, useful agents for gum production, and fortification ingredients to produce functional food. They are also well known to be a rich source of pigments, specifically xanthophylls [16] As a consequence, *Moringa leaves* are widely used as functional ingredients for the production of poultry feed, with documented, valuable effects on both hen health and egg quality [12,17,18]. Specifically, Abou et al. [17] studied how the supplementation of *Moringa leaves* may increase the egg quality of Rhode Island Red hens in terms of carotenoid yolk content. Other authors [18] have reported that the fortification of laying hen feed with *Moringa leaves* not only led to the production of eggs with an increased β-carotene content (+20%, *p* < 0.05, compared to the control) but also with a decreased cholesterol content (−20%, *p* < 0.05, compared to the control).

Goji berry (or wolfberry) is the common name for *Lycium barbarum* L., *Lycium chinense* L., and *Lycium ruthenicum* L. species, belonging to the Solanaceae family (eggplants) [19]. Due to their powerful chemical composition (polysaccharide, monosaccharide, essential oils, vitamins, amino acids, mineral elements, carotenoids, and flavonoids), goji berries have shown relevant health-promoting effects in humans. In this regard, some of the described beneficial activities included strengthening the immune system, regulating blood sugar levels, preventing vision loss, antioxidants, cholesterol-lowering, anti-bacterial qualities, and antifungal effects [19,20]. Recently, this precious fruit has also been proposed for the formulation of poultry feed to enhance the rate and the quality of the produced eggs [21]. Specifically, Duru et al. [21] reported that the supplementation of hen feed with goji berry leaves led to the production of egg yolk with significantly lower cholesterol levels compared to the control.

Based on such considerations, the main objective of this study was to evaluate the potential of *Moringa oleifera* dried leaves (DML) as a sustainable feed supplement, either alone or in combination with dried goji berries (DGB). Specifically, the nutritional and functional properties of eggs obtained from different feed formulations were evaluated in terms of carotenoids, cholesterol content, and antioxidant activity.

## 2. Materials and Methods

### 2.1. Hens and Experimental Design

Forty Lohman Brown Classic laying hens (19 weeks of age) with a similar weight and genetic background were randomly housed in 4 cages, 10 hens for each cage, each one 7560 cm^2^ in area. Each cage contained the feeder (120 cm) and 3 nipple drinkers. For 10 weeks prior to the treatment, enrolled hens followed a conventional hen diet based on soybean flour (2.85 M Kcal/kg feed) according to NRC guidelines (1994) [22]. The main nutritional components are reported in Table 1. Subsequently, the hens followed a diet for laying hens for 2 weeks. Then, the experimental diet was formulated according to the following protocol: the first group (control) followed a basal diet; the second group G2 was fed with 5% DML (Dried Moringa Leaves) + 10% DGB (dried goji berries); the third group G3 received 3% DML + 7% DGB; the fourth group G4 was administered 15% DML fortified feed. The total study duration was 56 days, and eggs were collected every 2 weeks (T0: before the experimental diet, after 4 weeks, and after 8 weeks of supplementation). All hens were exposed to natural photoperiod and temperature. Room temperature was maintained at 20–22 °C, and the light cycle was 16 h of light and 8 h of darkness. Light intensity was approximately 10 lx in the central story. All procedures reported here were approved by the Institutional Committee on the Ethics of Animal Experiments (CVS) of the University of Naples Federico II under protocol no. 851/2018.

### 2.2. Carotenoid Extraction Protocol

After egg collection, yolks were manually separated from the egg white and lyophilized. The powder obtained was submitted to the carotenoid extraction procedure, according to Hidayat et al. [23] with slight modification. Briefly, 1 g of the lyophilized samples was treated with 5 mL of ethanol/acetone mixture (3:2), homogenized for 5 min by ultra-turrax (T25-digital, IKA, Staufenim Breisgau, Berlin, Germany), and mixed on an orbital shaker (Sko-DXL, Argolab, Carpy, Italy) at 300 rpm for 15 min. Then, samples were placed in an ultrasonic bath for 10 min before being centrifuged at 6000 rpm for 10 min. The supernatants were collected and stored in darkness at 4 °C. The pellets obtained were re-extracted with 5 mL of the same mixture of solvents. Finally, the extracts obtained were filtered on syringe filters (0.45 mm in RC, Phenomenex, Torrance, CA, USA) and stored at −20 °C until the analysis.

### 2.3. Carotenoid Analysis by HPLC-DAD

HPLC-DAD analysis was performed with an HPLC system Jasco Extrema LC-4000 system (Jasco Inc., Easton, MD, USA), coupled with an autosampler, a binary solvent pump, and a diode-array detector (DAD). The chromatographic analysis was achieved according to the method described by Raffo et al. with slight modifications. The mobile phase consisted of acetonitrile (A) and EtOH/hexane/dichloromethane 1: 1: 1 *v/v* (B). The elution gradient used was as follows: 0–3 min 18% (B), 3–13 min 24% (B), 13–17 min 18% (B), 17–23 min 61% (B), and then the phase composition returned to the initial conditions in 5 min. The chromatographic column was a Gemini C18 (250 × 4.6 mm, 5 µm; Phenomenex, Torrance, CA, USA), with a flow rate of 1 mL/min. The carotenoids were detected at a wavelength of 450 nm. The quantitative analysis was conducted using a calibration curve of each standard with dilutions in the concentration range of 0.001–0.1 mg/mL [24].

### 2.4. Cholesterol Extraction Protocol

The lipid extraction was carried out according to the method of Bligh and Dyer (1959) [25] [NO_PRINTED_FORM]. A quantity of 3 g of lyophilized sample was treated with 20 mL of a solution (chloroform/methanol (2:1 *v*/*v*)) and stirred for 20 min. Then another 20 mL of the chloroform and methanol mixture were added and left in agitation for another 10 min. The sample was then poured into a separatory funnel and treated with 20 mL of 0.5% NaCl. At the end of this separation procedure, three different phases were obtained: the aqueous phase, solid phase, and chloroform phase. Only the chloroform fraction was collected, centrifuged, and evaporated to dryness by a rotary evaporator. The sample obtained was stored in the dark at −20 °C until the analysis.

### 2.5. GC-MS Sample Preparation

A quantity of 100 mg of lipid fraction was saponified with 2 mL of potassium hydroxide 2M in methanol solution according to Food Analytical Methods (2022) 15:1118–1131 1119 to liberate cholesterol. Unsaponified materials were extracted with n-hexane. The n-hexane extracts were injected, and cholesterol was quantified using the GC-MS technique.

### 2.6. Cholesterol Analysis by GC-MS

Quali/quantitative cholesterol analyses were performed according to Fernández et al. [26] with slight modifications. Gas chromatographic analyses were carried out on a gas chromatograph Agilent mod. 7890A (Agilent Technologies, Glostrup, Denmark) equipped with a mass selective detector Agilent mod. 5975C (Agilent Technologies, Glostrup, Denmark). A GC software MassHunter Acquisition B07.05 was used to control GC, while data were analyzed with MassHunter Quantitative Analysis B.07.01 (Agilent Technologies). Chromatographic separations were carried out using an HP-5MS Ultra Inert column (30 m × 0.25 mm i.d., 0.25 μm) from Agilent Technologies (Santa Clara, CA, USA). One microliter of the sample was injected in the split mode (5:1) at 280 °C. The carrier gas was helium at 1 mL and min^−1^. The oven temperature was programmed as follows: 220 °C at the beginning before being subsequently increased to 270 °C at the rate of 15 °C/min, then ramped up at 2 °C/min to 280 °C and maintained for 2 min, then ramped up again at 5 °C/min to 290 °C and held for 3 min. The total run time was 15.3 min. The temperature of the transfer line and the ion source was 295 °C. The electron multiplier voltage was 1000 V. A solvent delay time of 3 min was set. Mass detection was performed in the select ion monitoring (SIM) mode. The ions selected for cholesterol analysis were (*m*/*z*): the ions 275.3 and 301.3 as qualifier ions and the ion 386.4 as a quantification ion.

### 2.7. Antioxidant Activity

#### 2.7.1. DPPH^•^ Radical Scavenging Assay

The radical scavenging ability of the antioxidants present in the sample was evaluated by using the stable, radical 2,2-diphenyl1-picrylhydrazyl (DPPH). The analysis was performed by adding 100 µL of each sample to 1000 µL of a methanol solution of DPPH (153 mmol L^−1^). The decrease in absorbance was determined with a UV–visible spectrophotometer (Beckman, Los Angeles, CA, USA). The absorbance of DPPH radical without antioxidants (the control) was measured as the basis. All determinations were in triplicate. Inhibition was calculated according to the formula: [(Ai − Af)/Ac] ×100, where Ai is the absorbance of the sample at t = 0, Af is the absorbance after incubation time (9 min), and Ac is the absorbance of the control (the same volume of extract was replaced by methanol) at time zero. The 6-hydroxy-2,5,7,8-tetramethylchroman-2-carboxylic acid (Trolox) was used as a standard antioxidant, and the results were expressed in µmol of Trolox Equivalent (TE). Furthermore, the results were also reported as EC50, which is the amount of antioxidant necessary to decrease the initial DPPH^•^ concentration by 50% [26,27] The percentage variation (PV), with respect to the control, was calculated for each sample according to the following formula:

$$\%\mathrm{PV}=\frac{\left[(\mathrm{mmol}\;\mathrm{TE}/g\;\mathrm{of}\;\mathrm{sample}\;\mathrm{DW})]-[(\mathrm{mmol}\;\mathrm{TE}/g\;\mathrm{of}\;\mathrm{CTR}\right)}{\left[(\mathrm{mmol}\;\mathrm{TE}/g\;\mathrm{of}\;\mathrm{CTR})\right]}\times 100$$

#### 2.7.2. ABTS^•^ Radical Scavenging Assay

The method is based on the ability of antioxidant molecules to quench ABTS^•+^ radical (2,20-azinobis((3-ethylbenzotiazoline-6-sulfonate)), a blue-green chromophore with characteristic absorption at 734 nm. The assay was performed according to the method described by Babbar et al. [26] with slight modifications. ABTS solution was prepared by dissolving 2.5 mL of ABTS 7.0 mM solution and 44 µL of potassium persulfate 140 mM solution, which was left to react for at least 7 h at 5 °C in the dark. Then, ethanol-water was added to the solution until an absorbance value of 0.700 (0.05) at 754 nm was measured (Jasco Inc., Easton, MD, USA). The assay was performed by adding 1 mL of diluted ABTS working solution to 100 µL of the sample. The determination of the sample absorbance was accomplished after 2.5 min of reaction. All determinations were in triplicate. Blank was performed with ethanol in each assay. Inhibition was calculated according to the formula: [(Ai − Af)/Ac] × 100, (2) where Ai is the absorbance of the sample at t = 0, Af is the absorbance after 6 min, and Ac is the absorbance of the control at time zero. Trolox was used as a standard antioxidant. The results were expressed as both the µmol of TE and EC50, which is the amount of antioxidants necessary to decrease the initial ABTS^•+^ concentration by 50% [28]. The percentage variation (PV), compared to the control, was calculated for each sample according to the formula above.

### 2.8. Statistical Analysis

The results were analyzed using the SPSS 27.0 software (IBM corporation, New York, NY, USA) by using a one-way analysis of variance (ANOVA) followed by Tukey’s posthoc test. Significant differences were accepted at the 99% confidence level.

## 3. Results

### 3.1. Carotenoid Composition

The supplementation of the hen feed with different combinations of DML and DGB (G2–G4) led to a significant difference in terms of the carotenoid content in egg yolk. As reported in Table 2, a constant time-dependent increase of β-carotene content in the egg yolk was observed, reaching its maximum concentration after 4 weeks of treatment in the G3 and G4 groups (an increase of 181.38% and 116.01%, respectively). After 8 weeks of treatment, the β-carotene concentration increase, compared to the control, was maintained, and the highest concentration was achieved in G4 (1.74 mg/g). Xanthophyll accumulation in the egg yolk followed a different trend, with a maximum concentration reached after 8 weeks of treatment (Table 1). Lutein levels were highest in the egg yolk after 8 weeks in all the treatment groups, with an increase of 333.24% in G4, followed by 258.15% in G2, and 189.24% in G3. The same accumulation trend was followed by the other xanthophylls and analyzed, specifically, canthaxanthin and cryptoxanthin.

### 3.2. In Vitro Antioxidant Activity

In order to evaluate the effects of hen supplementation with DML and DGB on the functional properties of the eggs produced, the antioxidant activity of the hydroalcoholic extracts of egg yolks was evaluated by DPPH and ABTS tests. For both protocols, results were expressed as the mg of trolox equivalent/g of egg yolk DW, as shown in Table 3 and Table 4. Regarding the DPPH test, the highest increase in antioxidant activity compared to the control was observed after four weeks of supplementation with DGB, reaching a valuable increase of 61.46% compared to the control. After 8 weeks of treatment, the antioxidant activity of egg yolks slightly decreased (while still remaining higher than the control, +29.86%). The same results were obtained from the ABTS test; in fact, the maximal radical scavenging activity was found in the eggs laid by G4 group hens after 4 weeks of treatment (+45.41%). Considering both the protocols applied (DPPH and ABTS), the highest antioxidant activity was achieved after 4 weeks of supplementation with functionalized feed.

### 3.3. Evaluation of Cholesterol Content

In order to evaluate the potential functional effects of fortified feed, the cholesterol content in the collected egg yolks was analyzed. The most effective treatment was observed in eggs collected from the G3 group, with a valuable and significant decrease in cholesterol concentration of between −44.12% and −47.08% after 4 weeks and 8 weeks of supplemented nutrition, respectively, compared to the control (Table 5). The feed supplementation with DML (G4) showed a lower but still significant decrease in the cholesterol content of the egg yolks, while for the eggs collected from the G2 group, an insignificant decrease in cholesterol levels was observed.

## 4. Discussion

The biofortification of basal laying hen feed with natural matrices can improve the healthy potential of eggs produced without relying on artificial fortification [28]. From a functional point of view, two of the most studied parameters at the base of egg biological values are their carotenoid composition and cholesterol contents [9]. Various strategies have been studied to naturally modify these egg properties. Kotrbáček et al. explored the effects of supplementation on basal poultry feed with different concentrations of peculiar biomass and the freshwater algal *Chlorella* on the carotenoid content of hen egg yolks produced by Hisex Brown laying hens. They found that, after the experimental diet was followed by enrolled hens, the egg yolks contained higher carotenoid amounts compared to the untreated hens. Specifically, carotenoid concentration reached its plateau after the fourth experimental week, with an observed percentage increase ranging from +46% to +119% (*p* < 0.01) [29]. Other authors have fortified hen diets with different concentrations, such as (0, 150, 250, and 350 mg/kg of diet) marigold flower extract (MFE), a natural source of xanthophylls. Their results showed an increase in lutein and zeaxanthin content from approximately 11.5 to 5.9 mg/kg of dry matter, respectively, after the addition of 350 mg MFE per kg of feed [13].

In this context, our results are perfectly in line with the findings previously described showing a valuable increase of carotenoids, especially xanthophylls, in egg yolks produced by hens fed with experimental diets. As shown in the result section, the most relevant increase was observed after 8 weeks of experimental dieting in treatment group G4 (supplemented with only DML) of all the xanthophylls monitored (+317.89 for zeaxanthin, +333.24 for lutein, +311.85 for canthaxanthin, and +249.28 for cryptoxanthin), while the smallest evident increase was achieved by β-Carotene (+72.95). This accumulation trend was related to the different polarities of the two classes of carotenoids studied: xanthophylls and carotenes. The same was found by Na et al. [5], who studied the effect of the different supplementations of poultry feed using 50–300 mg carotenoids/kg feed with either β-8-apo-carotenoic acid ethyl ester (ACAEE), canthaxanthin, or β-carotene. They found that the trend of carotenoid accumulation in egg yolks was proportional to the polarity of the carotenoid used. In this regard, the higher accumulation of ACAEE (more polar) was followed by canthaxanthin and β-carotene (less polar) [5]. More specifically, among the xanthophyll compounds, the accumulation was proportional to molecular polarity, with the highest concentration increase observed for zeaxanthin in all the treatment groups, followed by lutein, canthaxanthin, and cryptoxanthin. These results reflect exactly the structural characteristics of these molecules, where zeaxanthin and lutein are more polar (hydroxylation of the benzyl group) than canthaxanthin and cryptoxanthin (the hydroxyl group was oxidated to ketone). Despite the eggs produced by hens belonging to the experimental group G4 showing the highest title of xanthophylls, a significant increase in these compounds were also found in G2 and G3 eggs. Reasonably, a higher increase in the total carotenoid content found in G4 due to both the higher carotenoid content in DML compared to DGB and the higher rate of feed supplementation (DML 15%) in G4. Nevertheless, the DGB feed supplementation for rabbits also resulted in a relevant increase of carotenoid and phenolic content in rabbit meat, while no data related to the hen feed fortification are available [30].

Considering the widely described antioxidant activity of carotenoids, and particularly xanthophylls [31], our data show that a more relevant radical scavenging activity was measured in samples collected from the experimental diet group G4, followed by G3, and at least G2.

Since the high cholesterol content in egg yolks is an inhibiting factor for consumers, reducing cholesterol in egg yolks will benefit the poultry industry and public health [18,32]. Thus, in this study, the second functional egg property monitored was the potential decrease of cholesterol content in egg yolks during the 8-week feeding trial. Specifically, the most relevant and significant cholesterol reduction was obtained in the egg yolk of hens fed with a fortified feed of DML (3%) and DGB (7%) after 4 and 8 weeks (group G3). These results show that the combination of DML and DGB has a synergic effect in decreasing cholesterol levels in egg yolks.

While the hen feed supplementation with DML has been largely described for its cholesterol-lowering effects, less documented is the effect of the goji berry supplementation on cholesterol content in egg yolks. In this regard, Bidura et al. reported that the fortification of hen feed with DML led to the production of eggs with a significantly lower cholesterol level in the egg yolk [18]. A supplementation of *Moringa leaves* may reduce the cholesterol content of egg yolks in two possible ways. On the one hand, it has been reported that the alkaloids contained in *Moringa leaves* are able to decrease lipogenic enzyme activity and increase the excretion of bile acids in feces [30]. On the other hand, the β-carotene contained in *Moringa leaves* may inhibit the hydroxymethyl glutaril-CoA enzyme, which plays a relevant role in mevalonic acid formation within the cholesterol biosynthesis pathway. Regarding the feed supplementation with goji berries, its addition to feeding improvements and rabbit meat quality decreased its cholesterol content. In addition, Duru et al. reported that the supplementation of hen feed with goji berry leaves led to the production of egg yolks with significantly lower cholesterol levels compared to the control [21] Reasonably, the G3 experimental diet has a synergic balance between DML and DGB intake, which results in a valuable cholesterol level decrease (−47% vs. the control). As described above, the carotenoids may decrease the egg yolk cholesterol content by inhibiting the hydroxymethyl glutaril-CoA enzyme, a key enzyme in the cholesterol biosynthesis pathway. On the other hand, a too-high xanthophylls concentration reached in G4 samples may activate another molecular mechanism responsible for insignificant cholesterol reduction observed in the G4 egg samples. Specifically, during egg formation, triacylglycerols, cholesteryl esters, and free fatty acids are synthesized in the hen liver and assembled to form egg-yolk precursors, such as VLDL (very low-density lipoprotein) and vitellogenin particles, which are transferred to the developing oocyte as key substances for the growth and development of embryos [33]. Gao et el. found that a high level of xanthophylls (G4) could linearly enhance the expression of the VLDL receptor (VLDLR) in the hen ovaries and consequentially increase the cholesterol and cholesterol-derivatives translocation from the liver to the embryos [34].

Finally, beyond the valuable egg functional properties studied, supplementation with a combination of DML and DGB may also have desirable health effects in laying hens. It was well-documented that the fortification of the hen diet by DML had a positive effect on the health status of laying hens, such as an increased expression of plasma antioxidant enzymes (glutathione peroxidase) [35]; a low concentration of plasma malondialdehyde (MDA) [35]; a high level of plasma total protein levels (index of efficient liver functionality); and low cholesterol serum level. For the DGB-based hen feed fortification, relevant hen plasma cholesterol-lowering effects were described [21].

## 5. Conclusions

In light of the promising results presented, an experimental diet based on the combination of DML and DGB may be considered an optimal hen feed for the production of potential “functional eggs”. Specifically, considering the obtained results, the perfect balance between the amount of DML and DGB was achieved in the G2 experimental diet group (3% DML and 7% DGB). Therefore, this feed formulation may be potentially applied by the agriculture industry in order to produce eggs with additional healthy properties. Furthermore, other studies are required to evaluate the additional potential effects of this supplemented diet, including an evaluation of hen health, the rate of egg production, and the potential improvement of their conservation.

## Figures and Tables

**Table 1:** Main nutritional components of hen basal diet.

Item	(% *w*/*w*)
Crude proteins	17.00
Crude fat	4.50
Crude fiber	3.91
Ash	14.50
L-Lysine	0.92
Methionine	0.40
Calcium	4.00
Sodium	0.13
Total Phosphorus	0.70

**Table 2:** Carotenoid composition of egg yolk of treated groups compared to the control.

		G1	G2	G3	G4	PV% G1 vs. G2	PV% G1 vs. G3	PV% G1 vs. G4
Zeaxanthin	T0	15.25 ± 0.84 ^a^	15.49 ± 0.50 ^a^	16.20 ± 0.69 ^a^	15.68 ± 0.76 ^a^	1.59	6.31	2.88
	4w	15.17 ± 0.27 ^a^	21.42 ± 0.61 ^c^	30.17 ± 0.85 ^d^	22.86 ± 0.85 ^d^	41.7	98.84	125.79
	8w	8.53 ± 0.45 ^b^	31.17 ± 0.87 ^d^	22.86 ± 0.85 ^c^	35.64 ± 0.52 ^d^	256.49	168.02	317.89
Lutein	T0	13.20 ± 0.39 ^e^	12.82 ± 0.85 ^e^	14.20 ± 0.71 ^e^	13.19 ± 0.76 ^e^	−2.90	7.52	−0.10
	4w	12.86 ± 0.33 ^e^	17.04 ± 0.49 ^e,f^	20.15 ± 1.11 ^g,h^	34.25 ± 0.97 ^h^	32.49	99.36	127.23
	8w	6.97 ± 0.07	24.96 ± 0.27 ^g^	25.63 ± 0.66 ^f^	30.19 ± 0.72 ^g,h^	258.15	189.24	333.24
Canthaxanthin	T0	9.58 ± 0.8 ^i^	9.57 ± 0.64 ^i^	10.85 ± 0.37 ^i^	9.59 ± 0.47 ^i^	−2.35	10.69	−2.20
	4w	11.60 ± 1.89 ^i,m^	13.20 ± 0.43 ^m^	15.16 ± 0.87 ^p^	21.69 ± 0.42 ^p^	43.64	72.92	87.05
	8w	5.32 ± 0.27 ^l^	18.20 ± 0.45 ^n^	20.05 ± 0.93 ^o^	21.93 ± 0.40 ^p^	241.78	184.77	311.85
Cryptoxanthin	T0	3.028 ± 0.17 ^q,r^	3.038 ± 0.16 ^q,r^	3.34 ± 0.22 ^r,s^	3.03 ± 0.17 ^q,r^	0.32	10.32	0.32
	4w	2.96 ± 0.87 ^q,r^	4.25 ± 0.35 ^s,t^	6.33 ± 0.59 ^u^	6.85 ± 0.36 ^u^	43.64	114.28	131.76
	8w	1.95 ± 0.33 ^q^	5.99 ± 0.26 ^u^	4.89 ± 0.49 ^t^	6.82 ± 0.69 ^u^	206.66	150.35	249.28
β-Carotene	T0	0.63 ± 0.09 ^v^	0.635 ± 0.04 ^v^	0.71 ± 0.02 ^v,x^	0.722 ± 0.06 ^v,x^	0.46	13.43	14.34
	4w	0.62 ± 0.05 ^v^	0.90 ± 0.04 ^v,x,y^	1.74 ± 0.44 ^w,z^	1.34 ± 0.29 ^x,y,w,z^	45.48	181.38	116.01
	8w	0.701 ± 0.20 ^v,x,y^	1.51 ± 0.33 ^y,w,z^	1.23 ± 039 ^v,x,yw^	1.88 ± 0.29 ^z^	39.22	12.91	72.95

Data are expressed as mean value (mg component)/g DW of egg yolk) ± SD of three repetitions. Mean values with different superscript letters are significantly different by Tukey’s multiple comparison test calculated with (*p* < 0.01), both along the columns and the lines.

**Table 3:** Radical scavenging activity evaluated by DPPH method in egg yolk in respect to the control.

	G1	G2	G3	G4	PV% G1 vs. G2	PV% G1 vs. G3	PV% G1 vs. G4
T0	28.34 ± 0.25 ^a^	28.87 ± 2.42 ^a^	30.05 ± 1.96 ^a^	32.30 ± 2.22 ^a^	1.87	6.03	13.97
4w	65.63 ± 1.01 ^b^	79.15 ± 2.64 ^c^	76.45± 2.26 ^c^	106.02 ± 2.53 ^d^	20.56	16.45	61.46
8w	50.07 ± 1.96 ^e^	69.65 ± 3.56 ^f^	68.35 ± 3.37 ^f^	65.02 ± 1.35 ^f^	39.11	35.51	29.86

Data are expressed as mean value (mg Trolox equivalents (TE)/g DW of egg yolk) ± SD of three repetitions. ^abcdef^ Mean values with different superscript letters are significantly different in Tukey’s multiple comparison test calculated with (*p* < 0.01), both along the columns and the lines.

**Table 4:** Radical scavenging activity evaluated by ABTS method in egg yolk compared to the control.

	G1	G2	G3	G4	PV% G1 vs. G2	PV% G1 vs. G3	PV% G1 vs. G4
T0	63.57 ± 1.95 ^a^	69.89 ± 6.52 ^b^	71.98 ± 1.84 ^b,c^	75.62 ± 1.28 ^c^	9.94	13.23	18.96
4w	125.46 ± 3.68 ^d^	155.19± 5.12 ^e^	134.23 ± 4.50 ^d^	182.43 ± 4.45 ^f^	23.70	6.99	45.41
8w	91.09 ± 2.83 ^g^	110.30 ± 2.08 ^h^	103.67 ± 5.39 ^h^	119.43 ± 4.88 ^i^	20.76	13.81	31.11

Data are expressed as mean value (mg Trolox equivalents (TE)/g DW of egg yolk) ± SD of three repetitions. ^abcdefghi^ Mean values with different superscript letters are significantly different in Tukey’s multiple comparison test calculated with (*p* < 0.01), both along the columns and the lines.

**Table 5:** Cholesterol content of lyophilized egg yolks quantified by GC-MS.

	G1	G2	G3	G4	PV% G1 vs. G2	PV% G1 vs. G3	PV% G1 vs. G4
T0	156.55 ± 8.51 ^a,b^	139.44 ± 10.19 ^a,d^	146.27 ± 12.12 ^a,d^	140.57 ± 6.39 ^a,d^	−2.12	−0.19	−4.08
4w	175.64 ± 13.61 ^b,c^	149.62± 10.68 ^a,d^	98.13 ± 12.04 ^e^	142.03 ± 0.37 ^a,d^	−14.79	−44.12	−19.12
8w	183.51 ± 9.06 ^c^	146.66 ± 11.04 ^a,d^	97.11 ± 12.10 ^e^	124.73± 6.73 ^c,d^	−20.08	−47.08	−32.03

Data are expressed as mean value (mg of cholesterol/100 g DW of egg yolk) ± SD of three repetitions. ^abcde^ Mean values with different superscript letters are significantly different in Tukey’s multiple comparison test calculated with (*p* < 0.01), both along the columns and the lines.

## Data Availability

The data used to support the findings of this study are included in the article.
